# IL-9 neutralizing antibody suppresses allergic inflammation in ovalbumin-induced allergic rhinitis mouse model

**DOI:** 10.3389/fphar.2022.935943

**Published:** 2022-09-12

**Authors:** He Zhao, Zhaowei Gu, Yunxiu Wang, Meng Wang, Yue Zhan, Xin Zhao, Zhiwei Cao

**Affiliations:** ^1^ Department of Otolaryngology Head and Neck Surgery, Shengjing Hospital of China Medical University, Shenyang, Liaoning, China; ^2^ Medical Research Center, Shengjing Hospital of China Medical University, Shenyang, Liaoning, China; ^3^ Department of Key Laboratory of Research and Application of Animal Models for Environmental and Metabolic Diseases, Shengjing Hospital of China Medical University, Shenyang, Liaoning, China

**Keywords:** allergic rhinitis, IL-9, Th2 cells, TSLP, JAK/STAT

## Abstract

Allergic rhinitis is mainly mediated by IgE after specific individuals are exposed to allergens. It is a common nasal mucosa disease of non-infectious chronic inflammatory disease and is often accompanied by asthma and conjunctivitis. In the study of allergic asthma, it was found that IL-9 participates in the pathogenic development of asthma. Because asthma and allergic rhinitis have the same airway and the same disease, it is inferred that IL-9 may also play an important role in allergic rhinitis. BALB/c mice received intranasal stimulation of ovalbumin (OVA) treatment at different times. The nasal mucosa of the mice were then sliced and stained with Sirius red and Toluidine blue, and eosinophils and mast cells in the mucosa were counted. ELISA was used to detect the expression of OVA-IgE in peripheral blood. The Th2 cell fraction in the mouse spleen was detected by flow cytometry. The expressions of IL-4, IL-5, IL-9, and IL-13 and their mRNA in mucosa were detected by real-time PCR and flow cytometry bead array analysis. Finally, the expression changes of Thymic stromal lymphopoietin related proteins and its mRNA, JAK1/2, and STAT5 proteins were detected by real-time PCR and Western blot. After the intervention with the IL-9 neutralizing antibody, the symptoms of allergic rhinitis in mice were significantly reduced. The expression of OVA-IgE in the peripheral blood of mice was inhibited, the fraction of Th2 cells in the spleen decreased, the related cytokines (IL-4, IL-5, and IL-13) were inhibited, and their functions decreased. The TSLP-OX40/OX40L signal pathway and JAK1/2-STAT5 signal are inhibited. IL-9 neutralizing antibody has a good therapeutic effect on the mouse model of allergic rhinitis, which may be related to the TSLP-OX40/OX40L pathway and JAK1/2-STAT5 signaling.

## Introduction

Allergic rhinitis is mainly mediated by immunoglobulin E (IgE) after specific individuals are exposed to allergens. It is a common nasal mucosa disease of non-infectious chronic inflammatory disease. It is mainly characterized by continuous sneezing, nasal congestion, nasal itching, and a clear, water-like runny nose. It is often accompanied by asthma and conjunctivitis (Bousquet et al., 2020). The incidence rate of allergic rhinitis is high and it seriously impacts the quality of life for people worldwide. It affects 10%–20% of the world’s population (Brozek et al., 2010) and has become a common global health problem that has attracted worldwide attention. Over the past 20 years, increasing attention has been paid to allergic rhinitis, which is often accompanied by allergic asthma. They are often defined as “the same gas, the same disease” (Grossman, 1997), and they affect each other. Although allergic rhinitis does not pose a threat to life, it has many adverse effects on quality of life of a person, economic burden, work, and emotion (Meltzer, 2016; Wise et al., 2018; Dierick et al., 2020).

Historically, the pathogenesis of allergic rhinitis has been a focus of research. It is recognized that the classical pathogenesis is the immune response caused by the imbalance of Th1 and Th2 mediated by Th2 (Romagnani, 2000). When Th2 is dominant, it causes epithelial cells and vascular endothelial cells to release inflammatory mediators such as leukotriene and histamine. They stimulate sensory nerve endings and the blood vessels of nasal mucosa, excite the nasal parasympathetic nerve, and then cause the vasodilation of the nasal mucosa and increase gland secretion. This results in symptoms such as nasal itching, sneezing, and a runny nose.

With the exploration of the mechanism of allergic rhinitis and the thorough understanding of Th cells (Wan, 2010), there has been an increase in awareness of increasing cytokines and Th cell subtypes. Although the imbalance of Th1 and Th2 cells can explain a variety of allergic diseases, the whole mechanism cannot be attributed to the imbalance of Th1 and Th2 cells. In previous studies (Reisinger et al., 2005) it was found that the enhancement of Th1 response has no negative impact on the allergic response dominated by Th2. It is, therefore, insufficient to explain the pathogenesis of allergic rhinitis simply by the imbalance of Th1 and Th2. It is clear that allergic rhinitis is a complex, slow, and non-infectious disease involving multiple mechanisms.

IL-9 is one of many cytokines that provide a new direction for the basic research of the pathogenesis of allergic rhinitis. Initially, it was thought that IL-9 was produced by Th2 cells, a growth factor of mast cells, and participated in the pathogenesis of allergic diseases. However, further research has led to an increased awareness of Th9 cells, a subset of helper T cells secreting IL-9. In a study of allergic asthma, it was observed that IL-9 participates in the pathogenesis and development of asthma (Koch et al., 2017; Seumois et al., 2020). In previous experiments, we verified the therapeutic effect of IL-9 neutralizing antibody on a mouse model of allergic rhinitis which is described (Gu et al., 2017). In subsequent research, we continued to use IL-9 neutralizing antibody to neutralize IL-9 in the mucosa and explore its pathways and mechanisms.

## Mice, materials, and methods

### Experimental mice

Female BALB/c mice aged 6–8 weeks were used in this experiment. They were obtained from Beijing Huafukang Bioscience Co. Inc. (Beijing, China). The mice were raised in an SPF environment for 12/12 h in a light/dark cycle, and they could freely obtain food and water without OVA. The experimental design was approved by the ethics committee of the Shengjing Hospital of China Medical University, No. 2020PS541K.

### Stimulation of allergic rhinitis mouse model

According to the methods, the mice were randomly divided into four groups, each comprising an allergic rhinitis group, a control group, an IL-9 neutralizing antibody group, and a homotypic control group, with 20 BALB/c female mice in each group (*n* = 80). From days 1–15, the allergic rhinitis, IL-9 neutralizing antibody, and homotypic control groups were injected with 0.1 ml of sensitizer intraperitoneally every other day, and the control group was injected with 0.1 ml of normal saline eight times. The sensitizer was ovalbumin (OVA; Sigma, United States) + aluminum hydroxide (Al(OH)_3_; Damao, Tianjin, China) + normal saline solution, in which the concentration of OVA was 1 mg/ml and the concentration of Al(OH)_3_ was 20 mg/ml. On days 16–26, the allergic rhinitis, IL-9 neutralizing antibody, and homotypic control groups had 10 μl nasal drops dropped into each nostril every day, and the normal control group had 10 μl of normal saline into each nostril every day, 11 times. The nasal drops consisted of OVA (5 mg/ml) + normal saline. The IL-9 neutralizing antibody and homotypic control groups were given 10 μl of nasal drip of IL-9 neutralizing antibody (eBioscience, San Diego, CA, United States) and IL-9 isotype negative control antibody (isotype Ab for anti-IL-9) (eBioscience, San Diego, CA, United States) 30 min before OVA. The doses of IL-9 neutralizing antibody were according to the previous study (Gu et al., 2017).

### Symptomatic counting and specimen preparation

Within 10 min after the last OVA challenge, an observer with unclear grouping recorded the number of sneezes and nose rubs per mouse. The mice were euthanized 30 min after the last OVA challenge. Before being euthanized, they were anesthetized with isoflurane (Sigma-Aldrich, St. Louis, MO, United States), the eyeball blood was taken and centrifuged, and the serum was reserved for ELISA. After removing the skin and mandible, the heads of the first five mice in each group were fixed in 4% paraformaldehyde. For the remaining 15 mice in each group, the nasal cavity was incised along the midline of the maxilla and the mucosal tissue in the nasal cavity and paranasal sinuses was removed with micro tweezers, placed into the cryopreservation tube, and immediately placed in liquid nitrogen. These mucosa would be used for real-time polymerase chain reaction (real-time PCR), CBA, and Western blot. There was very few nasal and sinus mucosa in each mouse, and therefore, the mucosa of a single mouse could not used for all the experiments. Consequently, the reserved mucosa was used for all the experiments according to each mouse. Five mice in each group were selected. After removing the spleen, they were immediately immersed in 1 ml of RPMI on ice and used for flow cytometry analysis.

### Eosinophils and mast cells count

In order to evaluate the degree of eosinophils and mast cells infiltration in the nasal mucosa, the nasal tissue was decalcified and embedded in paraffin. Coronal sections were made at a distance of approximately 5–8 mm from the nasal vestibule, with a section thickness of 4 microns. The sections were stained with Sirius red and Toluidine blue, and the eosinophils and mast cells in each region were counted under a 400X microscope.

### ELISA of serum specific ovalbumin-immunoglobulin E

The mouse peripheral blood serum, after centrifugation, was stored in a refrigerator at −80°C. The serum of five mice in each group was thawed at room temperature and the ELISA test of OVA-IgE of mouse serum was conducted according to the steps prescribed in kit instructions.

### The mRNA expression in mucosa of IL-4, IL-5, IL-13, IL-9, TSLP, TSLPR, IL-7R, OX40, and OX40L by real-time PCR

Total RNA was extracted from five randomly selected mucosa using RNAiso plus (Takara, Dalian, China). Genomic DNA (gDNA) was removed and complementary DNA (cDNA) was synthesized using the PrimeScript RT reagent Kit (Takara, Dalian, China). The TB Green^®^ Premix Ex TaqTM II was used to prepare the reaction system, and the Roche Lightcycle 480 II Sequence Detection System (Roche, Basel, Switzerland) was used for the amplification reaction to obtain the Ct value of the gene. The relative expressions of IL-4, IL-5, IL-13, IL-9, TSLP, TSLPR, IL-7R, OX40, and OX40L were calculated through normalized to *β*-actin. All the primer sequences used in the above experiments are shown in Table 1.

**TABLE 1:** Primers used in this study.

Gene	Primers
IL-4	F:TACCAGGAGCCATATCCACGGATG
	R:TGTGGTGTTCTTCGTTGCTGTGAG
IL-5	F:CTCTGTTGACAAGCAATGAGAC
	R:GTCTAGCCCCTGAAAGATTTCT
IL-9	F:AATGCCACACAGAAATCAAGAC
	R:ACACGTGATGTTCTTTAGGACT
IL-13	F:GCATTGAAGCAGTGGGCTCT
	R:GGCAGACAGGAGTGTTGCTC
TSLP	F:ACTGCAACTTCACGTCAATTAC
	R:CGAACTTAGCCCCTTTCAAATC
TSLPR	F:CTTGAGCCTGGAGTTCCGTTAT
	R:CTGCCTAGCCTTAAACACCAT
IL-7R	F:GAAGCCAAAAACGAGTCTGAAT
	R:ATCATTGGGCAGAAAACTTTCC
OX40	F:GGGATACTCTATGTCATCCGTG
	R:CTGCAGACAGTATCCTGAGTAG
OX40L	F:TGAGAATCTGGAAAACGGATCA
	R:TTCTGCACCTCCATAGTTTGAT
Actin	F:GCAGAAGGAGATTACTGCTCT
	R:GCTGATCCACATCTGCTGGAA

List of abbreviations used: F, forward primer; R, Reverse primer. All sequences start at 5’.

### Cytometric bead arrays of IL-4, IL-5, IL-13, and IL-9 in mucosa

Five reserved mucosal specimens were randomly selected, frozen on ice, and weighed. Then, 19 μl PBS was added to every 1 mg of sample. The samples were ground with a grinding rod until there were no obvious particles, centrifuged at 4°C, 13,000 r/min for 20 min, and then the centrifuged supernatant was sucked into a new EP tube for CBA. The flow cytometry bead array flex Kit (BD Biosciences, San Diego, California, United States) was used according to the manufacturer’s instructions to detect IL-4, IL-5, IL-9, and IL-13 levels in the supernatant.

### Western blot for Thymic stromal lymphopoietin, TSLPR, IL-7R, JAK1/2, and STAT5 in the mucosa

Total protein was extracted from the mucosa of the remaining five mice in each group using RIPA lysing buffer (Beyotime, Shanghai, China). The concentration of the extracted protein solution was determined using the BCA protein analysis kit (Beyotime, Shanghai, China). Total protein was separated by 10% sodium dodecyl sulfate polyacrylamide gels (SDS-PAGE). Each swimming lane had 30 μg of total protein added at the best concentration, and the result was transferred to the PVDF membrane (Beyotime, Shanghai, China). TSLP, TSLPR, IL-7R, JAK1/2, STAT5, and GAPDH were immunoblotted with a primary rabbit polyclonal anti-TSLP Ab, anti-TSLPR Ab, anti-IL-7R Ab, anti-JAK Ab, anti-STAT Ab (Abcam, Cambridge, MA, United States), and anti-GAPDH Ab (Affinity Biosciences, OH, United States), respectively. The PVDF membrane was then immunoblotted with secondary anti-rabbit IgG HRP (Affinity Biosciences, OH, United States). The blots were visualized using FGSuper Sensitive ECL Luminescence Reagent (Meilunbio, Dalian, Liaoning, China) and a Luminous instrument (GE Healthcare, Little Chalfont, Buckinghamshire, United Kingdom). The Western blot was analyzed quantitatively by ImageJ software from the National Institutes of Health, Bethesda, United States. The relative expression of the target protein was obtained by referring to GAPDH.

### Analysis of Th2 cell subsets in the spleen by flow cytometer

The previously reserved spleen was ground with a grinding rod until there was no large particle suspension, and the single-cell suspension was obtained after filtration with a filter screen. The above cell suspension was extracted to a sterile 24-well plate, and GolgiStop (BD Biosciences, San Jose, CA, United States) reagent was added to each reaction hole, and then incubated in a special constant temperature incubator at 37°C and 5% CO_2_ for 6 h. After it was removed, BV421-A: CD3 and FITC-A: CD4 antibodies (BD Biosciences, San Jose, CA, United States), were added, mixed well, and incubated in darkness for 15 min. Then, 1 ml of cell fix-lysing buffer was added, shaken gently, and mixed evenly. It was then incubated in the dark incubator for 20 min. Then, 1 ml of permeabilization buffer was added, shaken gently, mixed evenly, and placed into a special incubator at 37°C, where it incubated in the dark for 15 min pe-cy7-a: IL-4 antibody (BD Biosciences, San Jose, CA, United States) was added according to the instructions, mixed well, and incubated in darkness for 30 min. Then, 200 μl of cell staining buffer was added, and they were then analyzed on a BD Calibur flow cytometer. FlowJo VX software (FlowJo, Ashland, OR, United States) was used to analyze Th2 subsets by CD4^+^IL-4^+^.

### Statistical analysis

The data were collected by SPSS 20.0 software (SPSS Inc. Chicago, IL, United States) for the statistical analysis. A one-way ANOVA analysis compared multiple groups, and a Tukey test compared two groups. Spearman or Pearson correlations analyses were used to explore the relationship between symptomatic counting and metrics. A statistical difference was found when the set *p* value was less than 0.05. GraphPad Prism 8 software (La Jolla, CA, United States) was used to develop graphs of the statistical results.

## Results

### Symptomatology, eosinophils infiltration, mast cells infiltration, and ovalbumin-immunoglobulin E changes in peripheral blood of mice

**FIGURE 1:**
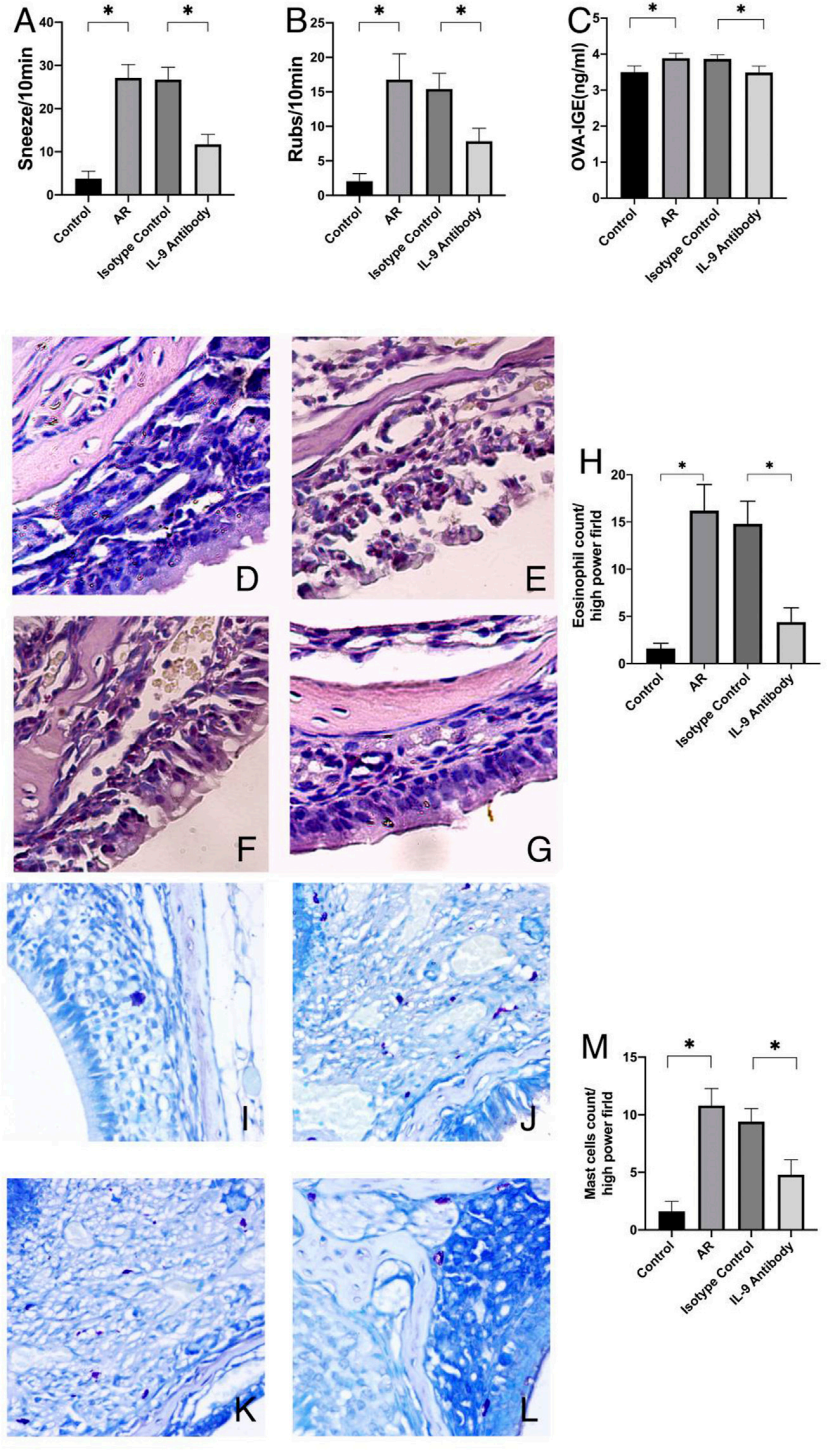
Symptoms and mucosal histology changed. AR was the allergic rhinitis group; IL-9 Antibody was the IL-9 neutralizing antibody group; Isotype control was the homotypic control group. **(A)** and **(B)** was the symptoms of the mice. After using IL-9 neutralizing antibody, the frequency of nasal rubbing and sneezing decreased. Every mouse has a rubbing and sneezing count of the 80 mice. One way ANOVA/Tukey was used for statistical analysis. All results were mean ± SEM. *p < 0.05. **(C)** was the data of protein expression of OVA-IgE in serum which was measured by ELISA of OVA-IgE of mice. The data are a representation of five independent experiments with duplicate samples. One way ANOVA/Tukey was used for statistical analysis. All results were mean ± SEM. *p < 0.05. **(D–F)** and **(G)** are pictures of Sirius red stained section under optical 400X microscope. **(D)** was the control group, E was the allergic rhinitis group, F was the homotypic control group, and G was the IL-9 neutralizing antibody group. H was the eosinophil counts of each group which counted by 400X microscope. The average number of eosinophils in each region was calculated in each Sirius red stained section (n = 5). **(I–K)** and **(L)** are pictures of Toluidine blue stained section under optical 400X microscope. **(I)** was the control group, **(J)** was the allergic rhinitis group, K was the homotypic control group, and **(L)** was the IL-9 neutralizing antibody group. **(M)** was the mast cell counts of each group which counted by 400X microscope. The average number of mast cells in each region was calculated in each Toluidine blue stained section (*n* = 5). One way ANOVA/Tukey was used for statistical analysis. All results were mean ± SEM. *p < 0.05.

After using an IL-9 neutralizing antibody, the sneezing and nasal scratching times of allergic rhinitis mice were significantly reduced, and the OVA-IgE of peripheral blood was also reduced (Figures 1A–C). In the pathological section, IL-9 neutralizing antibody significantly inhibited the eosinophils infiltration and mast cells infiltration in the mouse nasal mucosa (Figures 1D–M), which indicates the positive therapeutic effect of IL-9 neutralizing antibody on the mouse model of allergic rhinitis.

### IL-4, IL-5, IL-13, and IL-9 changes in the mucosa and Th2 cells changes in the spleen

**FIGURE 2:**
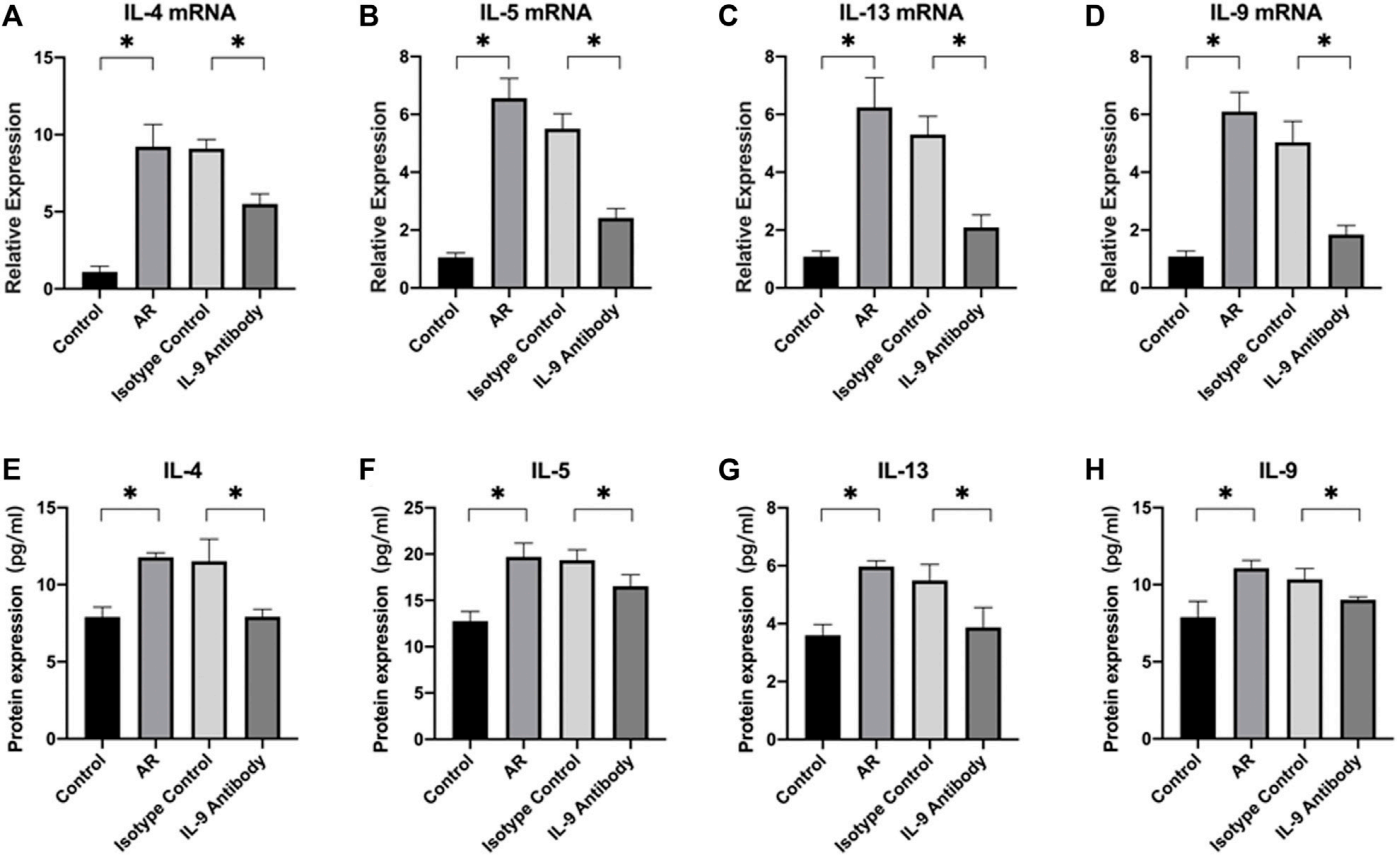
Expression of cytokines and their mRNA in nasal mucosa of mice in each group **(A)**–**(D)** The mRNA expressions levels of cytokines in nasal mucosa which were determined by real-time PCR. After total RNA was extracted, it was reverse transcribed into cDNA, and then real-time PCR was performed to obtain the Ct value of each gene. We used 2^−ΔΔCt^ to calculate the relative expressions. *β*-actin was used as the internal control. ΔΔCt = [ΔCt (Ct value of target gene in experiment group-Ct value of *β*-actin)-ΔCt (Ct value of target gene in control group- Ct value of *β*-actin)]. Each group repeats five independent samples, and each independent sample repeats three times to take the mean value. One way ANOVA/Tukey was used for statistical analysis. All results were mean ± SEM. *p < 0.05. **(E)**–**(H)** The protein expression of IL-4, IL-5, IL-13, and IL-9 in nasal mucosa of each group which was detected by flow cytometry bead array. Each group repeats five independent samples. One way ANOVA/Tukey was used for statistical analysis. All results were mean ± SEM. *p < 0.05.

**FIGURE 3:**
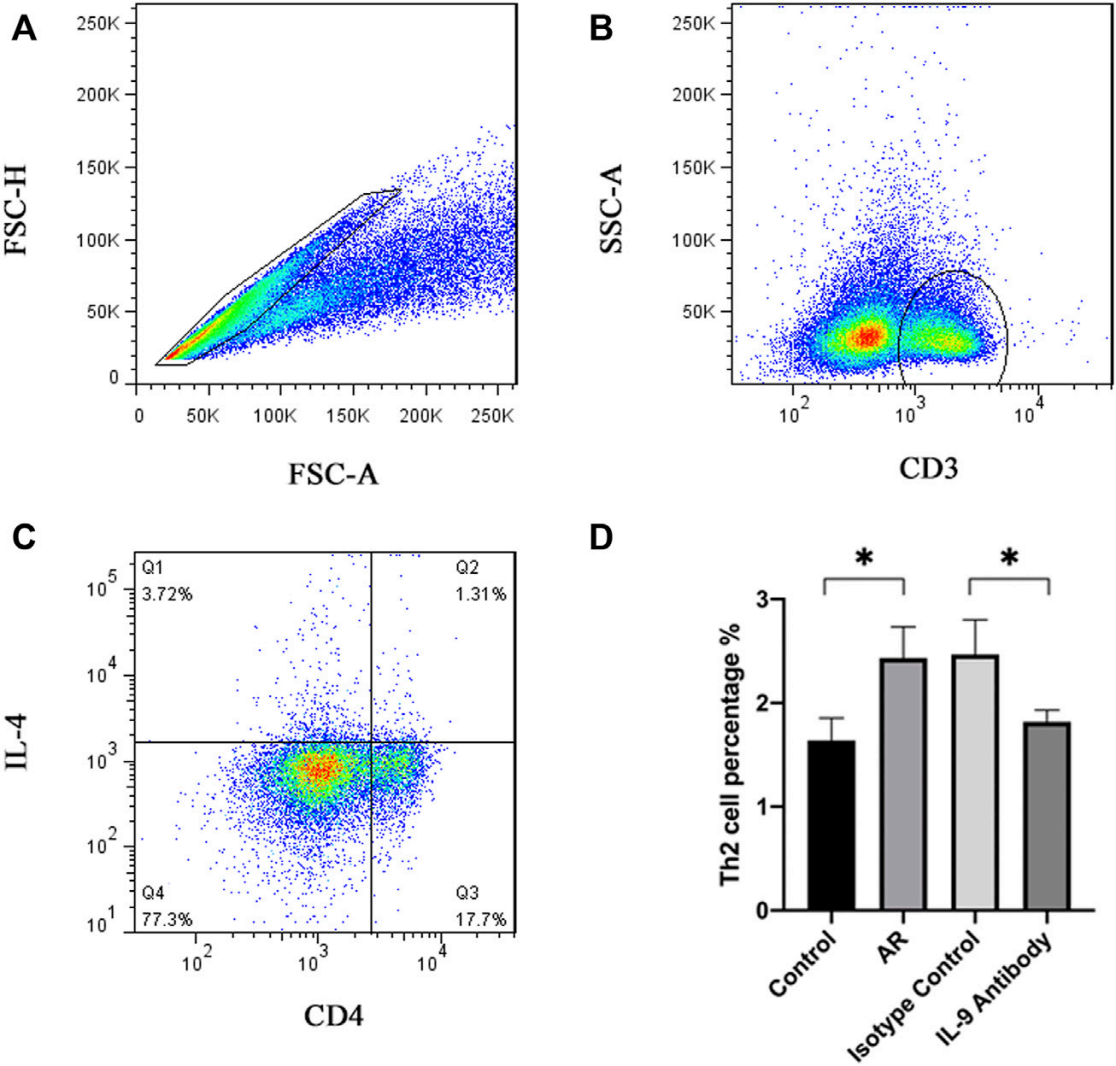
Schematic diagram and statistical diagram of flow cytometry. Figure **(A)**–**(C)** shows the Th2 delineation strategy of the control group, and other groups follow this scheme. Figure **(D)** shows the average number of Th2 cells in each group (*n* = 5). One way ANOVA/Tukey was used for statistical analysis. All results were mean ± SEM. *p < 0.05.

First, we used real-time PCR to detect the mRNA of IL-4, IL-5, IL-13, and IL-9 in the mucosa. We found that after using an IL-9 neutralizing antibody, the relative expression decreased (Figures 2A–D). Through the CBA, the protein expression of IL-4, IL-5, IL-13, and IL-9 decreased (Figures 2E–H). Through the detection of flow cytometry, Th2 cells in the mouse spleen were inhibited after using an IL-9 neutralizing antibody (Figure 3). IL-4, IL-5, and IL-13 were the cytokines of Th2 (Finkelman et al., 2010), and therefore, it was apparent that IL-9 neutralizing antibodies inhibited the Th2 cells and inhibited their cytokines, finally inhibiting the allergic reactions of the mouse model.

### Symptomatic counting correlation analysis

We next examined the correlations between symptomatic counting and metrics including eosinophils, mast cells, OVA-IgE, IL-4, −5, −9, −13, Th2 cells population.

Sneeze was significantly correlated with eosinophils (r = 0.936), mast cells (r = 0.911), OVA-IgE (r = 0.796), IL-4 mRNA (r = 0.904), IL-5mRNA (r = 0.910), IL-9mRNA (r = 0.837), IL-13mRNA (r = 0.876), IL-4 protein (r = 0.738), IL-5 protein (r = 0.861), IL-9 protein (r = 0.869), IL-13 protein (r = 0.876), Th2 cells population (r = 0.791).

Rubs was significantly correlated with eosinophils (r = 0.840), mast cells (r = 0.838), OVA-IgE (r = 0.723), IL-4 mRNA (r = 0.897), IL-5mRNA (r = 0.837), IL-9mRNA (r = 0.763), IL-133mRNA (r = 0.751), IL-4 protein (r = 0.745), IL-5 protein (r = 0.824), IL-9 protein (r = 0.781), IL-13 protein (r = 0.839), Th2 cells population (r = 0.783) (Table 2).

**TABLE 2:** Correlations across symptomatic counting and metrics.

		Eosinophils	Mast cells	OVA-IgE	IL-4 mRNA	IL-5 mRNA	IL-9 mRNA	IL-13 mRNA	Th2 cell	IL-4 protein	IL-5 protein	IL-9 protein	IL-13 protein
Sneeze	*r*	0.936	0.911	0.796	0.904	0.910	0.837	0.876	0.791	0.738	0.861	0.869	0.876
	*p*-value	0.000	0.000	0.000	0.000	0.000	0.000	0.000	0.000	0.000	0.000	0.000	0.000
Rubs	*r*	0.840	0.838	0.723	0.897	0.837	0.763	0.751	0.783	0.745	0.824	0.781	0.839
	*p*-value	0.000	0.000	0.000	0.000	0.000	0.000	0.000	0.000	0.000	0.000	0.000	0.000

Spearman or Pearson correlations analyses were carried out for all comparisons.

### Thymic stromal lymphopoietin-OX40/OX40L relative protein and mRNA expression changes

The relative mRNA expressions of TSLP, TSLPR, OX40, OX40L, and IL-7R in the nasal mucosa of allergic rhinitis mice were considerably higher than those in the control group. After using an IL-9 neutralizing antibody, the mRNA expression of TSLP, TSLPR, OX40, OX40L, and IL-7R decreased significantly compared with the allergic rhinitis group, and the difference was statistically significant. It showed that IL-9 neutralizing antibodies inhibited the mRNA expression of TSLP, TSLPR, OX40, OX40L, and IL-7R.

**FIGURE 4:**
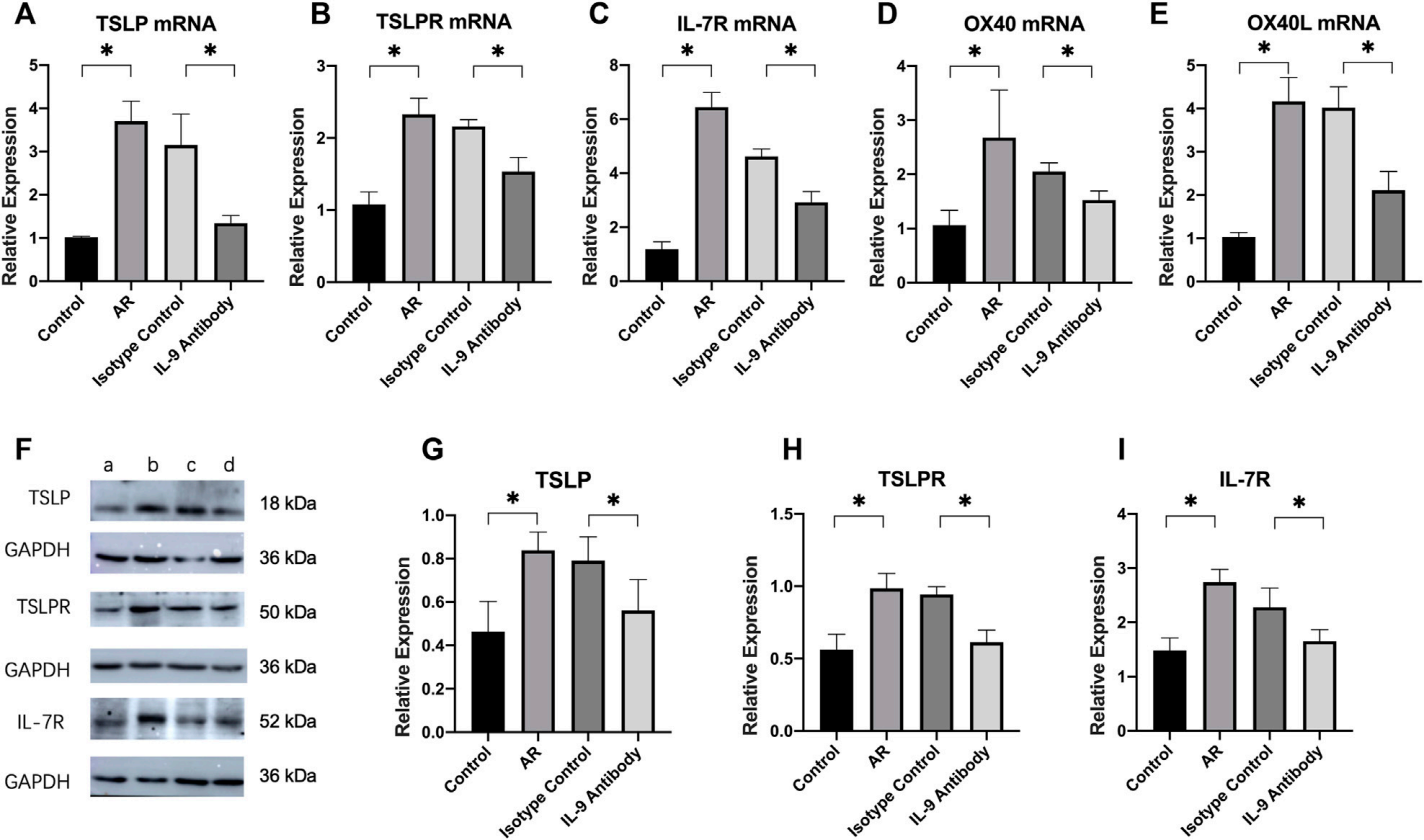
Expression of TSLP pathway and their mRNA in nasal mucosa of mice in each group. **(A)**–**(E)** Real-time PCR was used to detect the mRNA expressions levels of TSLP, TSLPR, IL-7R, OX40, and OX40L in nasal mucosa. We used 2^−ΔΔCt^ to calculate the relative expressions. We used *β*-actin as the internal control. Each group repeats five independent samples, and each independent sample repeats three times to take the mean value. One way ANOVA/Tukey was used for statistical analysis. All results were mean ± SEM. *p < 0.05. **(F)** Western blot analysis for TSLP, TSLPR, IL-7R in nasal mucosa. Each lane contains 30 μg of protein. Protein expression was normalized to GAPDH. a was the control group, b was the allergic rhinitis group, c was IL-9 neutralizing antibody group and d was the homotypic control group. Each lane is a representation of five independent experiment samples. **(G)**–**(I)** Western blot was analyzed quantitatively by ImageJ software. We used target/GAPDH to obtain the relative expression of the target protein. One way ANOVA/Tukey was used for statistical analysis. All results were mean ± SEM. *p < 0.05.

Using ImageJ to analyze the gray value of protein expression. The relative expression of the target protein was obtained by using the gray value of the target protein/the gray value of GAPDH, and then statistically analyzed. The relative expressions of TSLP, TSLPR, and IL-7R in the nasal mucosa of allergic rhinitis mice were significantly higher than those in the control group. The relative expression of these proteins was considerably lower than that in the allergic rhinitis group after using the IL-9 neutralizing antibody, and the difference was statistically significant (Figure 4). However, it remained higher than in the normal control group. These results show that IL-9 neutralizing antibody not only inhibits the expression of TSLP related pathway factor mRNA, but also inhibits the expression of protein.

### Phosphorylated JAK1/2-STAT5 protein expression changes

**FIGURE 5:**
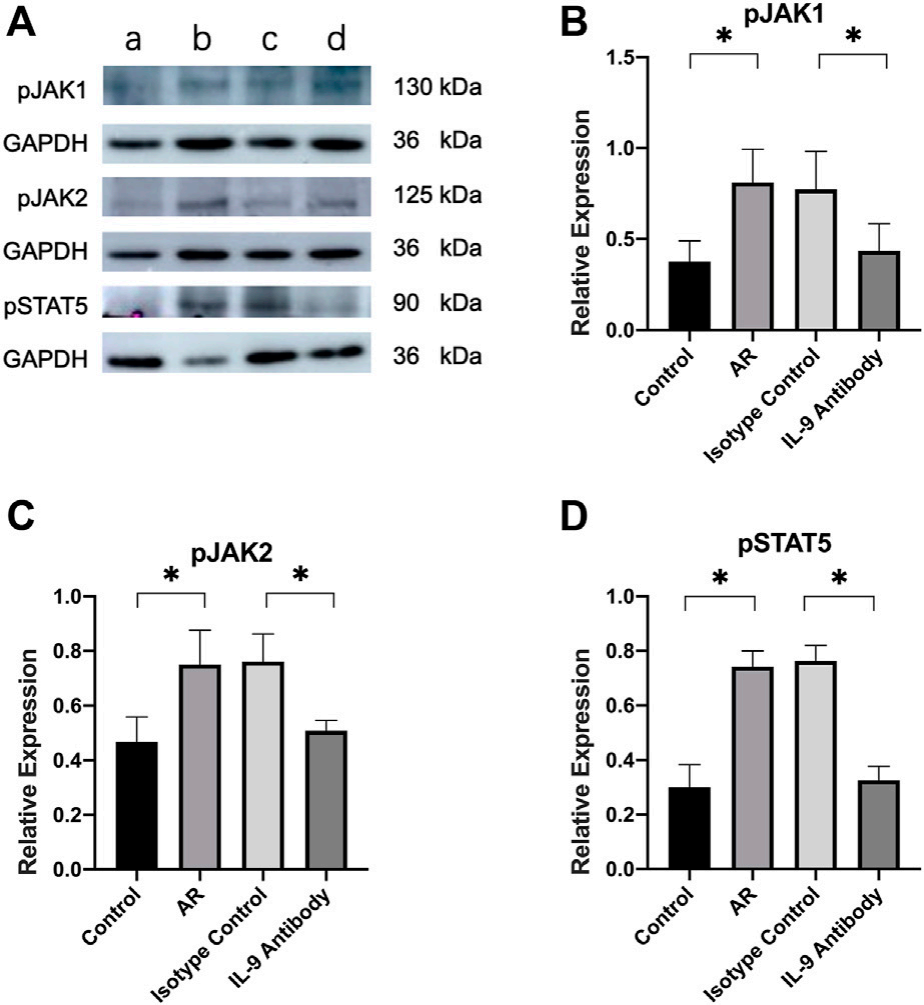
Expression of JAK1/2 and STAT5 in mucosa. **(A)** Western blot analysis for p-JAK1/2 and p-STAT5 in nasal mucosa. Each lane contains 30 μg of protein. The relative expression of the protein was based on GAPDH. a was the control group, b was the allergic rhinitis group, c was IL-9 neutralizing antibody group and d was the homotypic control group. Each lane is a representation of five independent experiment samples. **(B)**–**(D)** Western blot was analyzed quantitatively by ImageJ software. We used target/GAPDH to obtain the relative expression of the target protein. One way ANOVA/Tukey was used for statistical analysis. All results were mean ± SEM. *p < 0.05.

The relative expressions of phosphorylated proteins of JAK1/2 and STAT5 were also considerably higher than those in the control group. After using an IL-9 neutralizing antibody, the relative expression of these proteins was significantly lower than in the allergic rhinitis group, and the difference was statistically significant. However, it remained higher than in the control group (Figure 5).

## Discussion

Allergic rhinitis is mainly a type I allergic reaction dominated by Th2 mediated by IgE. It is mainly caused by the release of inflammatory mediators such as leukotriene and histamine from epithelial cells and vascular endothelial cells, which leads to the vasodilation of the nasal mucosa and increased gland secretion, resulting in symptoms such as nasal itching, sneezing, and runny nose (Bousquet et al., 2020). In recent years, with the detailed study of IL-9, it has been found that IL-9 can affect normal tissues and cells and exert its functions of anti-tumor and allergic reaction (Jones et al., 2012). IL-9 promotes airway inflammation through goblet cells, increases the secretion of IgE, and promotes the development of respiratory allergic inflammatory diseases (Gessner et al., 1993; Lauwerys et al., 2000; Lu et al., 2006; Xing et al., 2011). Therefore, IL-9 participated in the development and pathogenesis of allergic rhinitis. Consequently, we used an IL-9 neutralizing antibody to counteract IL-9, and then observed and investigated the important role of IL-9 in the mouse model of allergic rhinitis. IgE is mainly produced by plasma cells, which occur primarily in the lamina propria of the respiratory and digestive tract mucosa and is closely related to food allergy. The expression of IgE is positively correlated with allergic reaction (Rojas-Zuleta and Sanchez, 2017). Eosinophils and mast cells play an important role in allergic diseases such as asthma and allergic rhinitis (Temann et al., 2007; Fu et al., 2018). Therefore, the degree of eosinophil infiltration also reflects allergy severity (Temann et al., 2007; Zhao et al., 2013).

We found that the degree of eosinophils and mast cells infiltration in the nasal mucosa and OVA-IgE in the peripheral blood of allergic rhinitis mice were significantly improved after using an IL-9 neutralizing antibody, which confirmed that the IL-9 neutralizing antibody further improved the symptoms of allergic rhinitis by neutralizing IL-9 in the nasal mucosa of mice.

The main effector cells of cellular immunity are T lymphocytes. In the immune response, they produce cytokines that mediate inflammation and regulate other types of immune cells. Previous studies have shown that CD4^+^T cells have significant heterogeneity in cytokine expression, resulting in the discovery of cell subsets such as Th1, Th2, Th17, and Th9 (Veldhoen et al., 2008). After various stimuli, these Th cells secrete a class of small molecular proteins, namely, cytokines, to play numerous biological roles. These secreted extracellular cytokines regulate cell growth, differentiation, and function by binding to corresponding specific receptors, and finally play a biological role in regulating immune response. The functions of eosinophils and mast cells such as, migration and apoptosis, in AR are mainly regulated by cytokines secreted by Th cells (Wang et al., 2014). The immune response dominated by Th2 cells can explain various clinical symptoms caused by IgE in many cases of allergic rhinitis (Dardalhon et al., 2008; Veldhoen et al., 2008; Wong et al., 2010). Th2 cells participate in humoral responses by secreting cytokines such as IL-4, IL-5, and IL-13. IL-5 can stimulate the development of eosinophils in the bone marrow and their exit into the circulation, while IL-4 and IL-13 promote eosinophil infiltration through upregulated chemotactic factors such as eotaxin (Gu et al., 2017). IL-9 can act on CD4^+^T cells, including Th2 and Th17 cells, as a growth factor for T cells (Temann et al., 1998). In conclusion, we infer that IL-9 neutralizing antibodies affect Th2 cells by neutralizing IL-9, thus affecting the secretion of cytokines and finally inhibiting eosinophil infiltration and OVA IgE secretion, which play a positive role in allergic rhinitis in mice.

The spleen is an important immune organ, and we used flow cytometry to analyze the fraction of Th2 cells in the mouse spleen. We found that the Th2 cell fraction in the mouse spleen decreased after using an IL-9 neutralizing antibody, which was consistent with previous work (Gu et al., 2017). The simple reduction in cell fraction could not represent the decline of its function in allergic rhinitis. Therefore, we used CBA to detect the protein expression of IL-4, IL-5, and IL-13 in the mouse nasal cavity and paranasal sinus mucosa and real time PCR to detect the relative expression of their mRNA. We found that after using an IL-9 neutralizing antibody, the protein expression of IL-4, IL-5, and IL-13 decreased significantly, and their mRNA also showed a downward trend, which showed that the IL-9 neutralizing antibody further affected Th2 cells and cytokines of mice by neutralizing IL-9, which was consistent with previous work (Gu et al., 2017).

Thymic stromal lymphopoietin (TSLP) is mainly produced by non-hematopoietic cells such as epithelial cells, fibroblasts, and different types of stromal or stromal-like cells (Rawlings et al., 2004). In the immune system, dendritic cells are professional antigen-presenting cells that induce immune tolerance and immune activation. Dendritic cells express a variety of receptors, including TSLPR and IL-7R (Goswami et al., 2012). TSLP forms a new dimer after binding with TSLPR and IL-7R on the surface of dendritic cells, promotes JAK1 and JAK2 phosphorylation, further leads to STAT5 phosphorylation, and then plays its biological role (Yao et al., 2013; Liao et al., 2014; Olson et al., 2016). Further research on dendritic cells showed that dendritic cells expressed OX40L after being stimulated by TSLP and combined with the OX40 of primitive CD4^+^T cells to play a role, resulting in the increase of Th2 cell differentiation (Garo et al., 2017; Kaabachi et al., 2019). The TSLP pathway can promote the transformation of primitive naive CD4^+^T cells in lymph nodes into Th2 cells (Qiao et al., 2014), which explains the polarization of Th2. In the study of asthma, it was also observed that after inhibiting the TSLP-DC-OX40L signaling pathway, the secretion of Th2 cells and factors decreased, which played a therapeutic role (Purwar et al., 2012). In conclusion, we infer that IL-9 neutralizing antibodies are likely to play a role through the TSLP pathway.

Firstly, we detected the relative mRNA expression of TSLP, TSLPR, OX40, OX40L, and IL-7R in the nasal and sinus mucosa of allergic rhinitis mice by real-time PCR. After using an IL-9 neutralizing antibody, the mRNA expression of TSLP, TSLPR, OX40, OX40L, and IL-7R decreased significantly compared with the allergic rhinitis group. We simultaneously used WB to detect the relative expression of TSLP, TSLPR, and IL-7R and found that the expression of these proteins also showed a downward trend. The use of an IL-9 neutralizing antibody inhibited the TSLP signal pathway.

The JAK/STAT signaling pathway plays an important role in facilitating cell communication and controlling gene expression and is stimulated and regulated by a large number of internal environments in the body (Abdelilah et al., 2001; Kearley et al., 2011; Wang et al., 2019; Michelet et al., 2021). STAT plays a key role in the differentiation and effector function of almost all helper T cell subsets (Van Hulst et al., 2020). Its role in the TSLP signaling pathway cannot be ignored. Therefore, we used WB to detect the relative expression of JAK1/2 and STAT5 in the mouse nasal cavity and paranasal sinus mucosa and the relative expression after phosphorylation. After using an IL-9 neutralizing antibody, the expression of JAK1/2 and STAT5 decreased significantly compared to the allergic rhinitis group, and the degree of phosphorylation also decreased significantly.

In this study, after treatment with an IL-9 neutralizing antibody, the fraction of Th2 cells in the spleen of allergic rhinitis mice decreased, the expression of cytokines such as IL-4, IL-5, IL-13, and IL-9 in the corresponding nasal mucosa decreased, and its mRNA also decreased, indicating the anti-inflammatory effect of the IL-9 neutralizing antibody. Combined with the study of the TSLP signal pathway, it was speculated that the IL-9 neutralizing antibody reduced the secretion of TSLP by epithelial cells by neutralizing IL-9, leading to the decrease of TSLPR and IL-7R expression on the surface of dendritic cells, the decrease of dimer formation, the further decrease of JAK1/2 phosphorylation in dendritic cells, the decrease of STAT5 phosphorylation, the decrease of OX40L expression in dendritic cells, the decrease of OX40 expression in primitive CD4^+^T cells expressing OX40, the further decrease of polarization of primitive CD4^+^T cells into Th2 cells.

Although it cannot be confirmed that IL-9 neutralizing antibody regulated Th2 response through TSLP-OX40/OX40L signal pathway and JAK1/2-STAT5 signal, it was clear that the therapeutic effect of IL-9 neutralizing antibody was related to the above pathway. Local IL-9 intervention was used, but its effects on the systemic effect including blood, lung and lymph nodes were not excluded. It needs to be confirmed in future studies, which is the limitation of this study.

## Conclusion

IL-9 neutralizing antibody plays a therapeutic role in mice with allergic rhinitis, possibly through the TSLP-OX40/OX40L signal pathway and JAK1/2-STAT5 signal. This study is helpefull to further understand the pathogenesis of allergic rhinitis, and provided new ideas and basic theoretical support for the development of drugs for allergic rhinitis.

## Data Availability

The original contributions presented in the study are included in the article/supplementary material, further inquiries can be directed to the corresponding author.
